# Water Use Patterns of Sympatric Przewalski’s Horse and Khulan: Interspecific Comparison Reveals Niche Differences

**DOI:** 10.1371/journal.pone.0132094

**Published:** 2015-07-10

**Authors:** Yongjun Zhang, Qing S. Cao, Daniel I. Rubenstein, Sen Zang, Melissa Songer, Peter Leimgruber, Hongjun Chu, Jie Cao, Kai Li, Defu Hu

**Affiliations:** 1 College of Nature Conservation, Beijing Forestry University, Beijing, China; 2 Department of Ecology and Evolutionary Biology, Princeton University, Princeton, New Jersey, United States of America; 3 Smithsonian Conservation Biology Institute, National Zoological Park, Front Royal, Virginia, United States of America; 4 Altai Forestry Bureau, Altai, Xinjiang, China; 5 Wild Horse Breeding Center, Xinjiang Forestry Department, Urumqi, Xinjiang, China; University of Tasmania, AUSTRALIA

## Abstract

Acquiring water is essential for all animals, but doing so is most challenging for desert-living animals. Recently Przewalski’s horse has been reintroduced to the desert area in China where the last wild surviving member of the species was seen before it vanished from China in the1960s. Its reintroduction placed it within the range of a close evolutionary relative, the con-generic Khulan. Determining whether or not these two species experience competition and whether or not such competition was responsible for the extinction of Przewalski’s horses in the wild over 50 years ago, requires identifying the fundamental and realized niches of both species. We remotely monitored the presence of both species at a variety of water points during the dry season in Kalamaili Nature Reserve, Xinjiang, China. Przewalski’s horses drank twice per day mostly during daylight hours at low salinity water sources while Khulans drank mostly at night usually at high salinity water points or those far from human residences. Spatial and temporal differences in water use enables coexistence, but suggest that Przewalski’s horses also restrict the actions of Khulan. Such differences in both the fundamental and realized niches were associated with differences in physiological tolerances for saline water and human activity as well as differences in aggression and dominance.

## Introduction

In 2001, the Przewalski’s horse (*Equus ferus przewalskii*, henceforth referred to as “P-horse”) was reintroduced to Kalamaili Nature Reserve, Xinjiang, China (henceforth referred to as “KNR”) [1], where it was last sighted in China almost three decades ago [2]. For species used to be listed in the IUCN Red List as “extinct in the wild”, reintroductions become the last resort for establishing *in situ* populations[3] and not surprisingly each reintroduction receives attention worldwide. Following its reappearance in KNR, however, P-horses were faced with two major challenges: One was being able to cope with the severe environmental features at the release site, from which their ancestors disappeared several decades ago. The other was being able to cope with other resident wildlife species, especially the Khulan (*E*. *hemionus hemionus*) a closely related equid in KNR.

The first challenge tested the ability of P-horse to adapt to the harsh abiotic environment. KNR is part of the Gobi desert, where the summer is extremely hot and dry and the winter is extremely cold and snowy [4]. Although P-horse would need to cope with both extreme seasonal environments, we focused on summer conditions since other reintroductions to temperate steppe habitats have shown that P-horses can adapt to cold snowy conditions [5].

Although the Gobi desert was where P-horses were last sighted before their extinction in the wild [2, 6], modern feral and wild horses are typically found in mesic habitats, where food and water are plentiful during summertime [7], and survival and reproductive success is high [8]. Because such areas are also preferred by nomadic pastoralists and their large herds of livestock, the last surviving wild horses found in the desert were likely forced to survive at the edge of their historic range distribution [9]. Although this last population survived in these conditions, documentation on how well they coped with desert living was limited to anecdotal reports. Today P-horses have been reintroduced to a range of habitats, faring better in mesic ones than in the more arid areas where most reside [9]. While true desert-living animals cope with extreme heat and water stress via morphological, physiological and behavioral adaptations [10–12], P-horses as a more mesic adapted species are unlikely to have evolved structural or physiological traits for coping with desert-living. Nevertheless, modifications of behavioral strategies, such as social structure [13], daily rhythms [14], and movement patters [15], might allow them to adapt to these novel and extreme conditions. Van Dierendonck and de Vries suggest that P-horses can survive under arid conditions as long as they have continued access to water points [16].

P-horses, however, are not the only species requiring access to permanent water points in deserts. Before the arrival of the reintroduced P-horses, numerous other ungulates, carnivores, and small animals thrived in KNR. P-horse survival would therefore require the development of a water use strategy that facilitated co-existing with, or even the displacement of, other species, especially the abundant and evolutionarily closely related congeneric Khulan [17]. A recent genetic study showed that these two equid lineages only diverged from their common ancestor 4.0–4.5 million years ago [18]. Khulan phenotypically resemble a slightly smaller horse, with beige coat, slimmer limbs and “donkey” tail. Such similarities in phylogeny and phenotype, suggest the potential for a high degree of overlap in their fundamental niches, the full range of environmental conditions and resources an organism can possibly occupy and use [19]. With both species now living in sympatry and likely to share habitats and diets, the potential is also high for overlap in the realized niche, part of fundamental niche that an organism occupies as a result of limiting factors present in its habitat. When they last coexisted before efforts in conservation were instituted, P-horse went extinct in the wild.

Khulans and other *E*. *hemionus* subspecies are typical desert-living species with specialized feeding [20] and social strategies [21] facilitating resource acquisition in the desert. If the fundamental and realized niches overlapped with P-horses in the past and continue to do so today, then, the arid-adapted Khulans should be able to outcompete the mesic-adapted P-horses. However, fossils and historical records suggest that two equids were sympatric and coexisted for long periods of time [22, 23]. If they could have coexisted in the past then ancestral forms must have exhibited either minimally overlapping fundamental niches or carved out realized niches that were spatially [24] or temporally [25] segregated, thus significantly reducing interspecific competition [26]. Today, if the released P-horses or the native Khulans exhibit different fundamental niches or are able to adopt divergent realized niches, then coexistence would be fostered. If they fail to do so, however, then P-horses and Khulan, both “Endangered” species on the IUCN Red list [27], would be at risk since “complete competitors can not coexist” [17].

**Fig 1:**
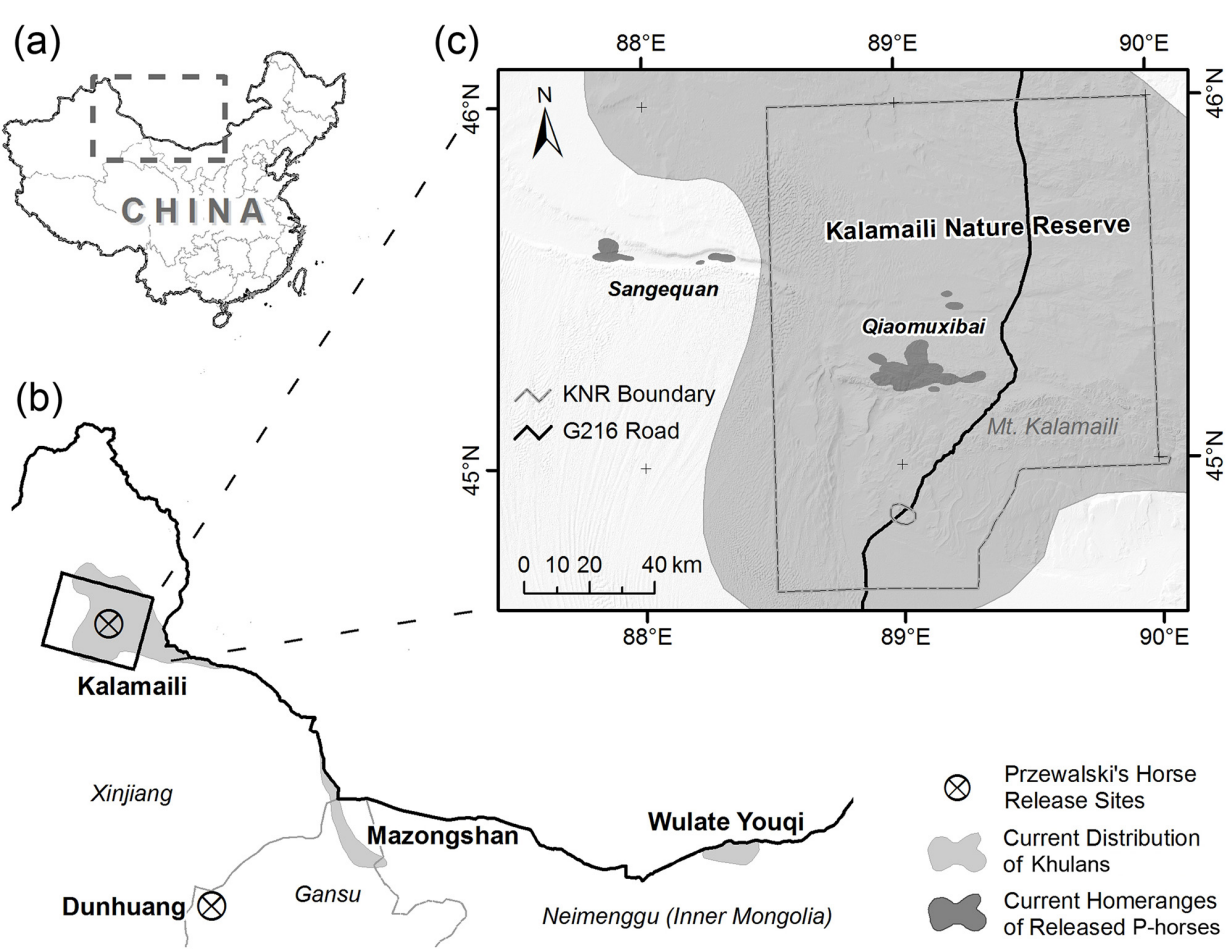
The current distributions of P-horse and Khulan in China. Regions near the western part of Sino-Mongolian border were highlighted by break lines (a) and zoomed into (b). The three extant Khulan habitats along the border were colored in light gray in (b), while two current release sites for P-horses were marked by circled “X”s. (c) showed the coverage of Kalamaili Nature Reserve and the distributions of P-horse (dark gray) and Khulan (light gray) in it. The distribution of P-horses was estimated by the 70% kernel home range of three collared harems (2007~2010).

In this study, we used camera traps to study the two equids’ behavioral strategies of summer water use. The study was carried out in Qiaomuxibai, the core of P-horse’s habitat in KNR and where the two equids share a common habitat and series of water points (Fig 1) from springtime to fall. Our study was carried out from 2010 to 2011, about a decade after the first group of P-horse was released in the Kalamaili area, and we presumed the behaviors and interspecific relationships of targeted species are now stable. Using camera traps reduced human interference on wildlife behavior and facilitated observations during both day and night and in difficult to navigate terrain [28–31]. Camera traps were deployed at water points during the dry season, when watering demand would be expected to be at its peak.

Water-use can also be affected by food availability as well as by the activities of other species, especially livestock and humans [32–34]. Yet, information on the watering patterns of P-horses and Khulans as impacted by interactions with each other as well as by the availability of water and its quality and human activity is extremely limited [28]. With both equid species again sharing a common desert landscape, our study is the first to systematically map the drinking behavior of these two species with advanced camera trap technologies and measure the level of interspecific competition over water that exists among these species. Understanding how each species copes with the presence of the other and what strategies, if any, are helping attenuate water competition between them will be essential for helping decision-makers design effective water management plans to maximizing the success of the reintroduced P-horse as well as the extant Khulan.

## Methods

### Study area

KNR is located in the northeastern side of the Dzungarian Basin, consisting of the Kalamaili Mountain Range (1464 m at its peak) and its northern extension (~700 m at the lowest point), where the landscape is predominately the Gobi desert and desert steppe. An arid continental climate is prevalent. Average annual temperature is 2°C, with an average of 20.5°C in July and −24.3°C in January [35]. Water is extremely limited. Average annual precipitation is low, 159.1 mm, with an average annual evaporation of 2090 mm [36]. Far from any ocean, humid air rarely reaches this landlocked region from spring until fall, resulting in limited and highly variable rainfall. In winter, however, westerly winds bring plenty of snow from the Atlantic, providing this area’s most important precipitation. No permanent surface runoff exists, and the only standing water comes from snow-melt, long-lasting natural springs, and temporary puddles (fed by rain or melt-water). Natural springs derive from underground water with most having been transformed into water points that provide the only reliable water during the dry season. The dry season starts in late June as puddles derived from the winter snow melt-water dry up, and ends around late October when the temperature drops and the first snows arrive.

The KNR was founded in 1982, with an area of 14,000 km^2^ (44°36' ~ 46°00' E and 88°30' ~ 90°03' N). This reserve was devoted to the conservation of endangered ungulates. It is one of the two places in the world [23] and the only place in China, where P-horse and Khulan are sympatric (Fig 1). In 2001 China released the first group of P-horse in KNR. By the end of 2013, this population had grown to 121 individuals, most of which were born in the wild. The population is currently divided into 15 harems and 1–2 bachelor groups. Meanwhile, the KNR housed the largest Khulan population in China. Before China banned private firearm ownership, illegal poaching and pastoral encroachment caused dramatic reductions of Khulan habitat [37, 38]. Khulan distribution was reduced from its historical range in northern Inner Mongolia and Xinjiang, to three isolated sites (Fig 1) along the Sino-Mongolian border [39]. After the firearm ban in early 1990s, Khulan population has been quickly recovering, at least in KNR, from a low of 358 individuals in 1985 [40] to 3128 ~ 4711 individuals in 2007 [41]. Besides the two equids, there are the Altai argali (*Ovis ammon ammon*), the goitered gazelle (*Gazella subgutturosa*), the grey wolf (*Canis lupus*), the fox (*Vulpes vulpes* and *V*. *corsac*), and various birds and small mammals living in the KNR. As a desert ecosystem, the vegetation wildlife rely on in KNR is limited. Most parts of the reserve have less than 10% vegetative cover [36], but a few oases can be found near the natural springs. The dominant plant species include shrubs (mainly *Haloxylon ammodendron*, *H*. *persicum*, and *Tamarix laxa*), forbs (*Anabasis salsa*, *Krascheninnikovia ceratoides*, *Artemisia desertorum*, *Reaumuria* songarica, etc.), and grasses (predominately *Stipa sareptana* and *Carex duriuscula*).

Despite being a water-poor desert, KNR is quite snowy in winter, which not only provides a continuous water source to desert-living creatures during the winter, but also attracts local herdsmen and their livestock into KNR. As the only sanctioned human land use inside the reserve, herdsmen stay from late November until mid-March. This generates conflict with wildlife over the limited non-snow covered vegetation. In the first winter after the initial P-horse reintroduction, several horses died due to food shortage, which to a large extent resulted from harassment by herdsmen and domestic horses [42]. This incidence forced the managers to corral the remaining horses during the winter when the herdsmen were present. To evaluate the feasibility of eliminating the need for winter corralling, beginning in 2013 two groups were allowed to roam free.

### Camera traps

**Fig 2:**
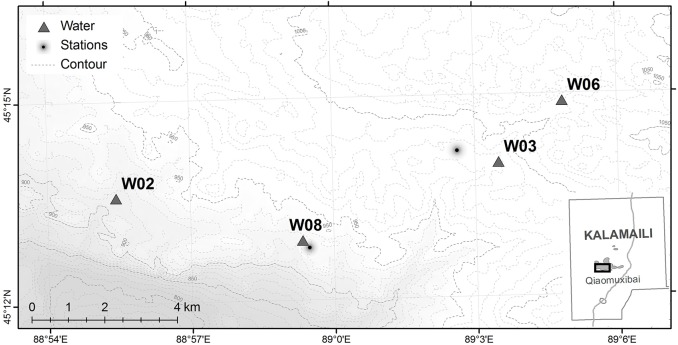
The geographic locations of the four permanent water points (dark triangles) and the two ranger stations (black halo dots) in Qiaomuxibai. The background was this area’s topographic map with contour lines.

Water points in KNR are the only permanent water sources for ungulates during the dry season. In the 1980’s, KNR converted natural springs into water points of 5–10 m in diameter to increase the availability of water for all of KNR’s wildlife. However, over the years some of these water points have become saline as water has evaporated and minerals have accumulated. During our study, four permanent water points provided the only dry season watering data (Fig 2). They were also the most important water points in this area, since others were ephemeral, of limited capacity, or were of high salinity or inaccessible. Besides differences in size and salinity, these four water points varied in use by humans, especially researchers (Table 1). Although no permanent residences or sustained human land is permitted in KNR during the dry season, reserve staff and researchers (5–7 people) used two base stations in Qiaomuxibai, which were close to some of the water points (Fig 2).

**Table 1:** The status of four permanent water points in Qiaomuxibai, with the measures of quality (salinity) and safety (distance to station), and the results from data collections in 2010~2011.

ID	Salinity(‰)	Distance to Stations (km)	Effective Camera Days	Number of Total Events	
				P-horse	Khulan
**W02**	5	5.5	100	400	998
**W03**	10	1.2	147	105	712
**W06**	11	3.2	156	86	2084
**W08**	6	0.2	161	465	55

At water points, we deployed passive infrared cameras (ScoutGuard SG-550, Boly Media Communications Co. Ltd., Shenzhen, China). According to the sizes of the water points, up to three cameras were used to permit full coverage of the water point and its bank. We blended the cameras into the environment by attaching them on natural stable objects, such as shrub branches or high rock piles. Most cameras were about 5 m from the water and at least 0.2 m above ground, as a tradeoff between a camera’s sensitivity and coverage. We avoided pointing them towards the sun or other cameras to reduce interference. After the cameras were set, their positions and compass bearings were recorded so that their positions are constantly maintained throughout the study period. Moreover, cameras were programed with a 30s delay between sequential photos for saving battery life and increasing efficient use of limited memory on SD cards. To identify the right delay that balances quantity and quality of data, a preliminary study was conducted with much shorter delays. We found most of the drinking bouts were longer than 30s. As the nature of equid group size, composition and stability, individual passing by the camera within 30s of the first individual was almost certainly from the same group. Even when there was an occasional missing count of those from other groups “passing by”, their likelihood of drinking was very low. Since this study only focus on the actual “drinking” of the animal, such occasion would not jeopardize the data’s overall accuracy. We checked all cameras twice per week, replacing memory cards and batteries and ensuring camera positions and settings remained intact.

### Data analysis

There were several factors limiting our analysis of the camera trap data. First, it was very difficult to identify individual animals from the images. Unlike equid species with highly variable coat patterns (e.g. feral horses and zebras), Asian equids lack obvious unique marks for identifying individuals. Second, it was difficult to estimate the actual number of unique individuals using any particular water point. While P-horses live in closed membership family groups [43], Khulan live in fission-fusion aggregations that tend to coalesce into large temporary herds at water points [13]. Larger herds tended to trigger more shots, but without the ability to identify individuals, it was almost impossible to know whether individual Khulan were captured in two successive pictures and thus double counted. And third, because the depth of field changed from day to night, images taken during the night might also have underestimated the number of water users. Although Scoutguard SG550 detect individuals up to 15 m, the flash can only light objects within 5–10 m at night. So even if events during day and night were triggered at the same distance, images at night would not necessarily include individuals between 10–15 m who would be captured during the day.

Given these difficulties, counts of individuals or estimations of group size were not possible. Instead, we modified O’Brian’s trapping event technique [44] to quantify a species’ visits to water points. An independent trapping event of water use was defined as consecutive photographs of individuals of a given species taken less than 5 min apart at the same water point. Given that the maximum length of a single drinking event by an individual or an entire group is five minutes, a five-minute capture interval was used to sample camera to insure that individuals captured from different trapping events were considered independent. We quantified each species’ water use frequencies, both hourly and daily, by counting the number of independent trapping events triggered during a given observation period. However, there were a few cases when Khulans arrived as a large herd, far beyond the capacity of the small water point to serve them all simultaneously. As a result the parade of individuals by the camera sometimes lasted for hours. Such events were rare; only 20 were recorded and they represented < 0.5% of the total events. Accordingly we treated the long continuous streaming as one single event when calculating the daily watering frequencies. When calculating the hourly time budget and percentages, however, we divided such long events into hourly segments corresponding to the clock hours. Because camera traps sometimes failed because of lost battery charge or because memory space was exceeded, the effective total trapping period was shorter than our total study period. Therefore, we only used ‘effective camera days’, the days when the camera operated continuously for at least 2/3 of a day (> 18 hours), when calculating average daily watering frequencies. A species’ daily watering frequency at a specific water point was derived by dividing total events recorded each ‘effective day’ at that water by the total number of each camera’s effective working hours during that day.

A species’ water visit may favor a specific time of day. To quantify such circadian preference on water usage, we defined a new index called Normalized Daytime Water Visitation Index (NDWVI) as:

$${NDWVI}_{i}\;=\;\frac{{Nday}_{i}-\;{Nnight}_{i}}{{Nday}_{i}+\;{Nnight}_{i}}$$

where *Nday*_*i*_ and *Nnight*_*i*_ were the numbers of water visits by each species during daytime (0600–1800) and nighttime (1800–0600) respectively within a unit time *i*. When NDWVI = 0, a species’ water point visits were considered indifference between day and night during the given period; if NDWVI > 0, day time drinking is preferred whereas when NDWVI < 0, nocturnal drinking is preferred.

All data were analyzed in R [45] and its external packages, *MASS* and *ppcor*. We first used Chi-squared test of Independence to examine whether there was any water point preference by each equid. Then the partial Pearson’s correlation test was applied to determine if particular features of water points such as salinity, distance to monitoring station, or visit frequency of other species were significantly associated with particular water point use by each equid species. To examine the correlation between two equid species’ circadian patterns of water use, the Pearson’s correlation test was applied. We used the Least-square estimation method in the linear/quadratic regressions and the Akaike information criterion (AIC) to select the regression models with the best fit to the circadian patterns. Moreover, a 10-day sliding window was used to calculate the NDWVIs of both species throughout the study period. Lastly, we identified all behavioral events within a single frame for both species. From these pictures we could count the number of successful drinking events, the number of times groups were seen waiting while other groups drank as well as other the number of vigilance, repelling or retreating events of each equid. Contingency tables of the proportionate differences in these activities by species were analyzed using the Fisher’s exact test. All comparisons employed 2-tailed tests with a significance level of 0.05, except in partial correlation tests, when we used the Bonferroni correction [46] and set the ‘family of tests’ significance level to 0.005 to reduce the chance of type I error. R package *ggplot2* was used for plotting.

### Ethics statement

This study was approved by the National Natural Science Foundation of China (Grant No. 30970545) and passed its ethical evaluations. It was also reviewed by Princeton University IACUC (Protocol No. 1835). The fieldwork was carried out under the authority of a scientific permit issued by Altai Forestry Bureau, Xinjiang, China.

## Results

During two dry seasons in 2010 and 2011, camera traps collected > 50,000 images over a period of 563 days. These images were divided into 6151 trapping events. Khulan and P-horse triggered 3385 (55.03% of all events) and 1056 (17.17% of all events) events respectively; they were the most common species using the water points (S1 Fig).

### Water point utilization and preferences

**Fig 3:**
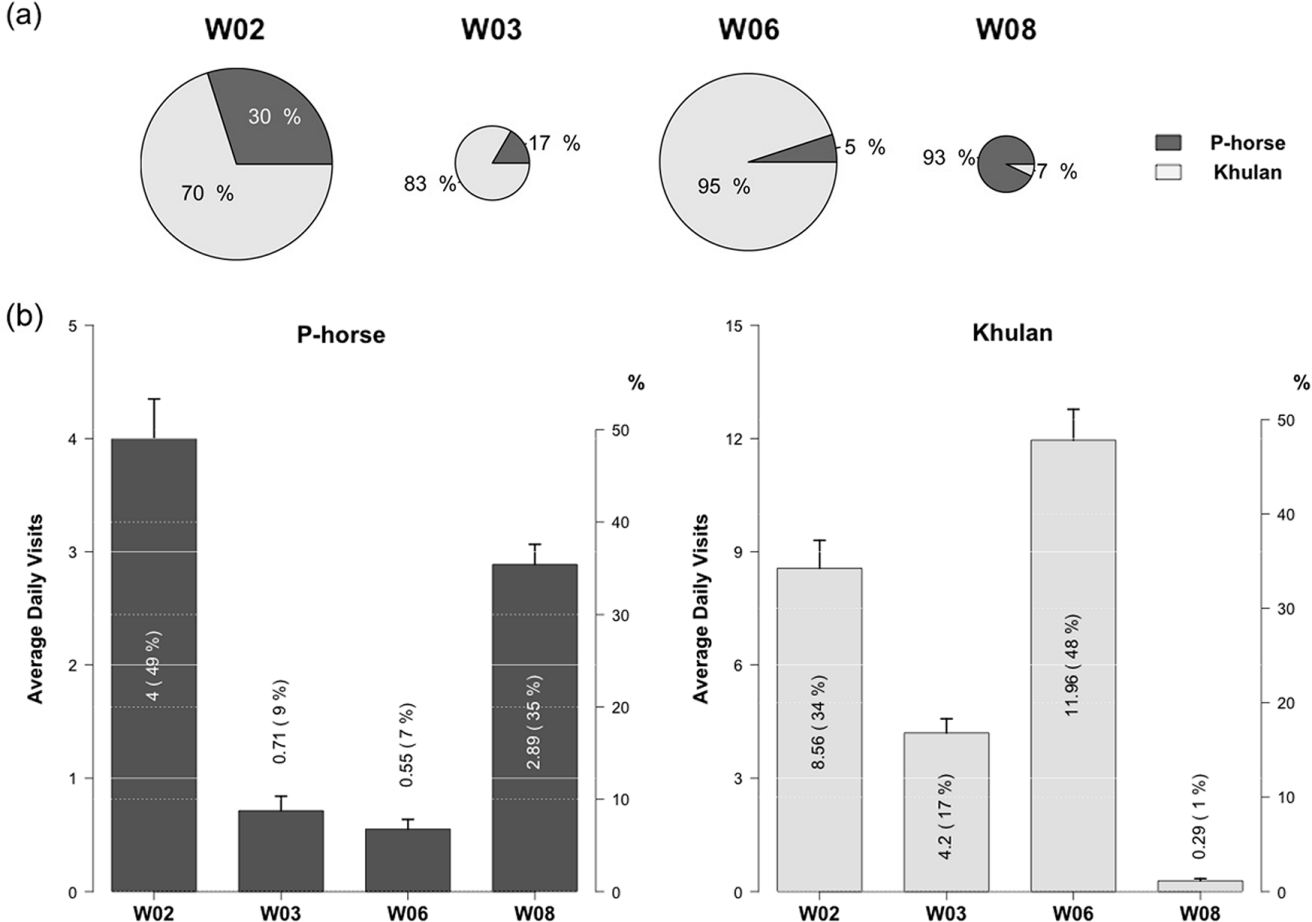
The percentages (a) and frequencies (b) of average daily water visits by P-horse and Khulan at four water points. The texts inside bars are the average number and percentage of water visits by each equid per day per water point. Error bars represented the standard error (*SE*).

Both species used all four water points in the study area, but based on the number of encounters detected by the cameras (Fig 3A), Khulan outnumber P-horses at three of them—W02, W03 and W06. P-horses only numerically outnumber Khulan at water point W08. Use, however, does not connote preference and as the proportion of encounters within each species captured by the cameras reveals, each species showed strong preferences for particular water points (Fig 3B; *Chi-squared* 393.566 [P-horse] and 1654.911 [Khulan], *P* < 0.001). P-horse groups were captured drinking almost 50% of the time at water point W02 and 35% of the time at water hole W08. Khulan groups, however, were seen drinking almost 50% of the time at water hole W06 and almost 35% of its time at W02. Clearly, water hole W02 is highly ranked and preferred by both species. Overall, however, the species showed disproportionate use of the different water points.

Variations in two factors could have contributed for these proportionate differences: salinity and in proximity to human influence (Table 1). W02 and W08 contained the highest quality water (salinity 5 ~ 6 ‰), while W03 and W06 had the lowest (salinity 10 ~ 11‰). But of the two low saline and thus high quality water points, W02 was further (5.5 km) from a ranger station than was W08 (0.2 km) (Fig 2). And of the two more saline water points, W06 was further from human activity (3.2 km) than was W03 (1.2 km). That the two equid species did not use all four water points in similar ways suggests that either differences in physiology, behavior or both may be responsible for the patterns of use.

Mesic-adapted P-horses showed strong preferences towards low saline water points (S2 Fig). 84.47% of the events recorded at W02 and W08 consisted of P-horse sightings, while the other two water points with high salinity were seldom visited by P-horses (Fig 3B). Partial correlation analysis measures the degree of association between two variables without the effects of controlled environmental variables. It showed that water use by P-horses was negatively correlated with salinity when controlling for human or Khulan factors (Table 2; Pearson’s, *partial R* = - 0.559, *P* < 0.0001), but showed no correlation with distance to ranger settlements when the other variables controlled (Table 2; Pearson’s, *partial R* = 0, *P* = 0.993).

**Table 2:** Partial Pearson’s correlation tests on factors might affect the water point visits of the two targeted equid species.

	Salinity		Distance		P-horse		Khulan	
	CC^a^	*P*	CC	*P*	CC	*P*	CC	*P*
**Salinity**	1	0						
**Distance**	-0.193^b^	3.28E-06***^c^	1	0				
**P-horse**	-0.559	3.03E-57***	0	0.993	1	0		
**Khulan**	0.402	2.71E-25***	0.483	6.14E-39***	0.190	4.86E-06***	1	0

The degree of association (correlation coefficient) between two variables was tested after removing the effects of other variables. Significant *P* values were marked with asterisks.

^a^Pearson’s Correlation Coefficient

^b^Strength of Correlation: <0.1 zero correlation; 0.1–0.3 weak correlation; 0.4–0.6 moderate correlation; 0.7–0.9 strong correlation; >0.9 perfect correlation

^c^Significance level after Bonferroni correction: ‘***’ <0.0001.

Because P-horse’s live in closed membership family groups or relatively stable bachelor units, we were able to identify daily drinking patterns of individual P-horses. Such sightings showed that P-horse groups throughout the dry period used the same water points for long periods of time, often for several months. Moreover, different groups rarely used the same water point simultaneously. When watering patterns of all P-horse groups were combined, the four water points were visited by P-horses collectively 8.15 times per day. In this region of KNR there were three harems (> 10 horses each) and one fairly stable bachelor group (4–11 bachelors). Thus each group drank on average approximately two times per day.

Whereas P-horses appear indifferent to human activity, Khulans appear to actively avoid drinking close to ranger stations. As noted above, Khulan groups disproportionately used water points W02 (34.23% of Khulan events), W03 (16.81%) and W06 (47.81%) more often than W08 (1.14%). Even though W08 was low in salinity, it was the water point closest to human activity (Table 1). The others were at least 1 km from nearby human base stations (S2 Fig and Table 1). After controlling for the effects of the other variables via partial correlation analyses, water use frequency in Khulan declined with increases in proximity to human settlements and salinity (Table 2; Pearson’s, *partial R* = 0.483 [distance] and 0.402 [Salinity], *P* < 0.0001).

Moreover, since all desert-living species should prefer low saline water, both equid species must preferentially frequent low salinity, high quality water points if freely able to do so. P-horses were sighted most often (84.47% of P-horse sightings) at both low salinity sites irrespective of distance to ranger stations. Khulans, however, only frequented the high quality site most distant from these stations (34.23% comparing to 1.14%). Yet at the two sites far from human disturbance, Khulans were sighted drinking 41% more often at the more saline site W06 than at the less saline site W02 (S2 Fig; Kruskal-Wallis, *W* = 6382.5, *P* < 0.05). Clearly, Khulan are drinking at high quality water sites much less than P-horses and the barrier preventing them from doing so cannot be solely the result of disturbance by people. Since there is a weak, but strongly positive correlation between two equids’ daily water visits to particular sites (Table 2; Pearson’s, *partial R* = 0.190, *P* < 0.0001), the extra barrier to moving freely is likely to be the presence of P-horses.

### Circadian rhythms

**Fig 4:**
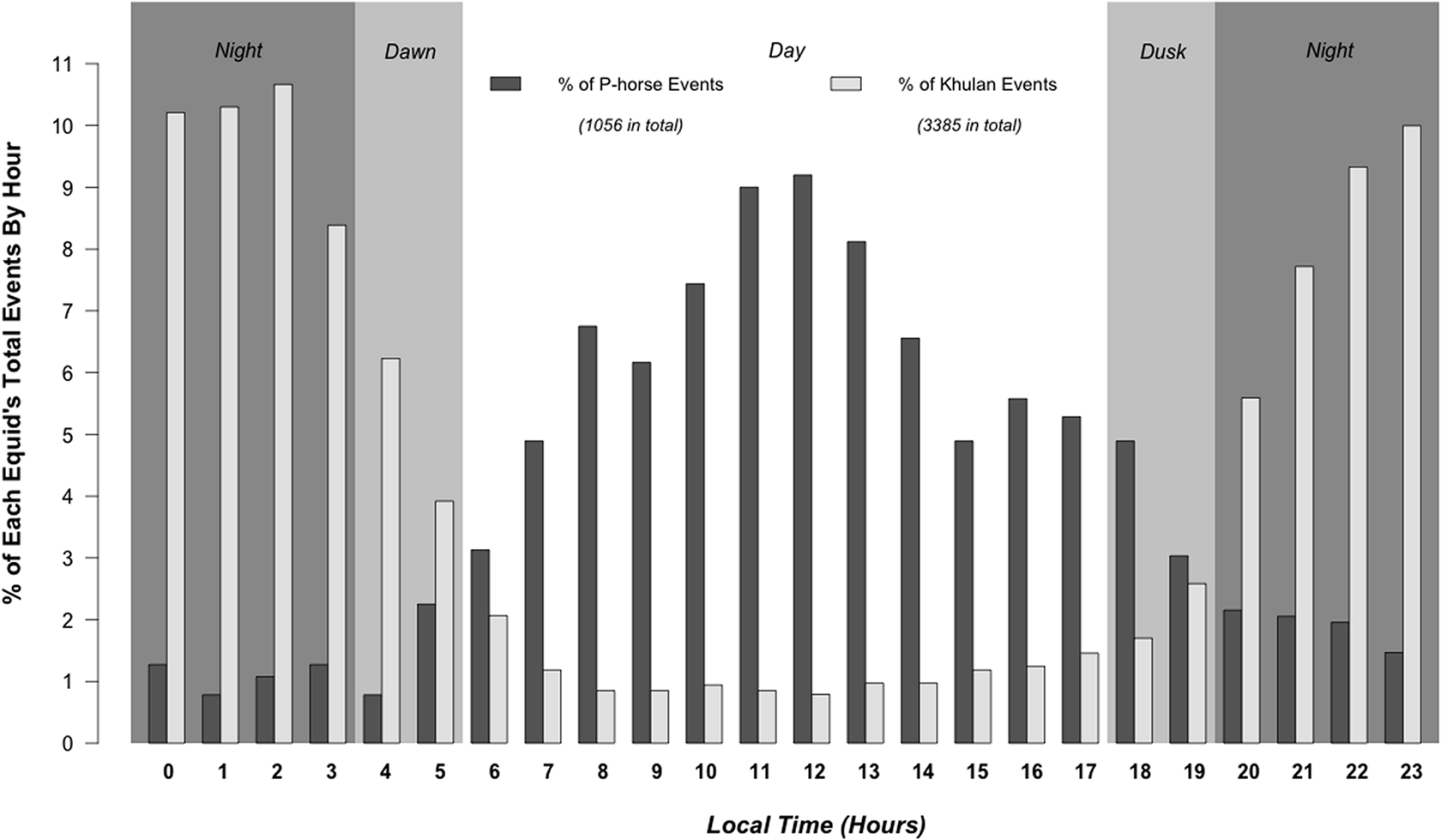
The circadian rhythm (by hour) of P-horse (dark gray) and Khulan (light gray). Each bar represented the percentage of total trapping events of that species recorded in that hour of day. Background colors indicated the day (white), twilight (light gray) and night (dark gray).

Despite the small positive correlation in water point visitations, the two equid species displayed largely non-overlapping uni-modal circadian water use patterns (Pearson’s, *R* = - 0.84, *P* < 0.001) based on percentage visits per hour to all water points (Fig 4). A behavior is considered diurnal or nocturnal if > 80% of its encounters captured on camera occurred during daylight hours (0600–1800) or nighttime hours (1800–0600) respectively [47]. Fig 4 clearly shows that water use by P-horse is strongly diurnal; 81.90% of the drinking events occurred during daytime. The P-horse daily rhythm followed a cosine trigonometric curve (*R* ^*2*^ = 0.91, *P* < 0.001), with its crest near noon and its trough near midnight. Conversely, Khulan tended to use water points at night; 84.93% of drinking events occurred during night or twilight periods. Their rhythm was uni-modal pattern, following a cosine-like pattern peaking at midnight (*R* ^*2*^ = 0.84, *P* < 0.001).

Such circadian water visiting preferences also had different intensities towards different water points. In general, each equid species’ circadian water use rhythm for a given water hole matched its overall water use rhythm (S3 Fig). As shown in Table 3, P-horse use of the two high-quality sites was largely diurnal (${\bar{NDWVI}}_{W02}\;$ = 0.55 and ${\bar{NDWVI}}_{W08}\;$ = 0.575), becoming less so for the lower quality sites that were also frequented much less often (${\bar{NDWVI}}_{W03}\;$ = 0.212 and ${\bar{NDWVI}}_{W06}$ = -0.113). Clearly water quality played an important role in their temporal water preferences. In contrast, Khulans used all water sites at night irrespective of quality or preference (Table 3), as all NDWVIs were lower than -0.7, but the degree of nighttime drinking was negatively correlated with distance to ranger stations (S1 Table; Pearson’s, *partial R* = 0.190, *P* < 0.001).

**Table 3:** Two equids’ average daily daytime (Nday) and nighttime (Nnight) visit frequencies at four water points and their average Normalized Daytime Water Visit Indices (NDWVI).

		$\bar{Nday}$	$\bar{Nnight}$	$\bar{NDWVI}$
**W02**	P-horse	3.080	0.954	0.550
	Khulan	1.345	7.264	-0.702
**W03**	P-horse	0.482	0.212	0.276
	Khulan	0.328	4.015	-0.862
**W06**	P-horse	0.232	0.232	-0.113
	Khulan	1.348	9.848	-0.840
**W08**	P-horse	2.297	0.484	0.575
	Khulan	0.000	0.266	-1.000

**Fig 5:**
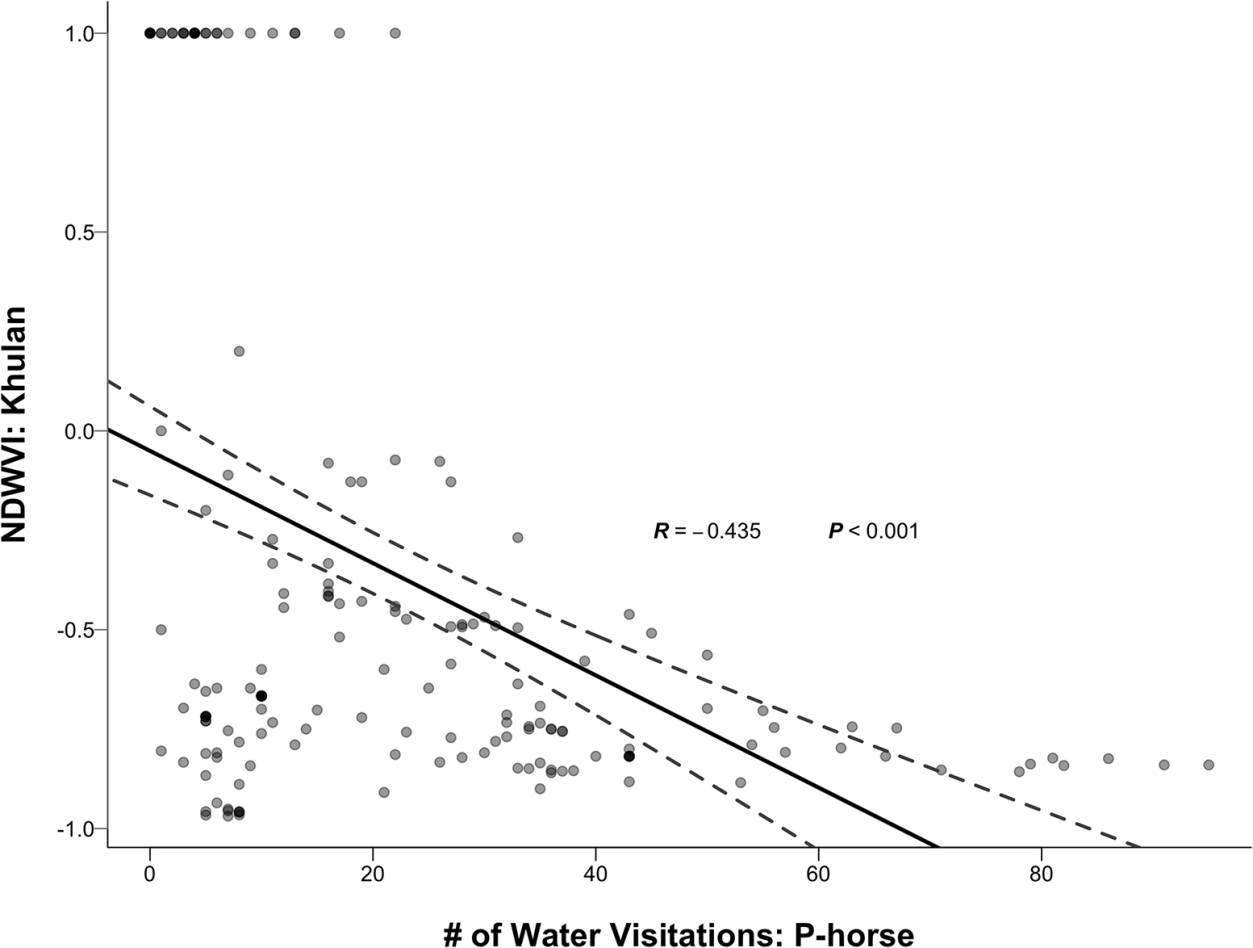
The number of water visitations by P-horse negatively affected the circadian pattern of water use (NDWVI) by Khulan at W02, the water point disproportionally used by both species. The grey-scaled scatter point represented each observation within 10-day sliding window. All scatter points were transparent so that overlapped points would be darker in the plot. A linear regression line (solid) was fit to the dataset with the 95% Confidence Intervals (dashed).

As previously noted, W02 was the only water point that both equids frequently used. Regression analysis of the presence of P-horse showed a strong negative impact on daytime water use by Khulan (Fig 5; *y* = −0.014 ∙ *x* + 0.050; Pearson’s, *R* = -0.435; *P* < 0.001). This suggests that P-horses act as a barrier preventing Khulan from drinking at water point W02 during daytime. The reciprocal analysis shows that increased use of water point W02 had no effect on the NDWVI of P-horse (*y* = 7.82*e* ^−4^ ∙ *x* − 0.547; Pearson’s, *R* = 0.095, *P* = 0.214).

### Evidence of competition

Although differences in their spatial (location) and temporal (circadian) water use patterns largely separated the two species with respect to drinking, there were 23 events clearly captured on camera when both equids were together in the same frame at the same water point (0.518% of 4441 total events captured on camera). When seen together in a frame we noted whether individuals of the two species drank simultaneously or whether individuals of one species stood back while individuals of the other drank. We also recorded whether or not waiting individuals were vigilant attending to the drinking behavior of members of the other species and whether or not individuals of one species retreated from the water in response to approaches from individuals of the other species (Table 4). Of the 23 events when both species were captured in the same frame, only 5 showed individuals of both species drinking water at the same time and place. In the other 18 instances, only P-horses were seen drinking. Overall, P-horses were seen drinking during all 23 recorded encounters when Khulans were present, whereas Khulan were standing and waiting during 15 out of 23 such episodes. In addition, Khulan were also captured retreating or being vigilant in 5 of the 18 events when only horses drank. P-horses were never seen standing or watching on the 5 occasions when Khulan drank. When seen together at water points, P-horse were free to drink when they wanted, whereas Khulan appeared to behave as subordinates in approximately 80% of encounters when at water points with P-horses.

**Table 4:** Contingency table of behavioral responses by two equids to each other during direct encounters at water points.

	P-horse N (%)	Khulan N (%)	Fisher’s exact test *p* value
*Successfully drinking*	23 (100)	5 (21.7)	2.387e-08*** ^a^
*Standing and waiting*	0	15 (65.2)	1.916e-06***
*Displaying retreating or vigilant behaviors*	0	5 (21.7)	0.0491*

Among 23 events two species were seen together in a frame, we noted whether the species drank simultaneously (the 1^st^ row), whether one species stood back while the other drank (the 2^nd^ row), and whether or not it was vigilant attending to the drinking behavior of the other species or whether or not one species retreated from the water in response to approaches from the other species (the 3^rd^ row).

^a^Significance level: ‘***’ <0.001; ‘*’ <0.05.

## Discussion

Using a common set of water points at the same time of year might suggest overlapping fundamental niches between P-horses and Khulan. Yet in our study, the two species showed differential use of common water points and used them at different time of day during the dry season. If the ability to divide the niche in both space and time is largely determined by intrinsic physiological differences associated with the capacity to cope with the hot and dry conditions it would be consistent with an ancient Chinese proverb: *mesic horse and arid donkey* suggesting that the two species have evolved distinct fundamental niches.

Support for this proposition comes from a variety of factors. First, spatial divergence emerged in part from differences in the two equids’ ability to use water differing in quality. In the desert, high quality water with low salinity is limited. Only half the water points we sampled had low salinity levels (< 6 ‰). They were also likely associated with high predation and competition since such water points were preferred by all wildlife including ungulates, wolves, and even humans and their livestock herds [23]. While true desert-adapted mammals may tolerate a range of water qualities because of specialized renal systems [48–50], mesic adapted species such as P-horses are likely physiologically constrained from doing so. In fact, P-horses appeared capable of surviving desert conditions only by drinking on average at least twice a day from low saline water points, a pattern not often seen in more mesic environments [21]. Khulans, however, were able to subsist by drinking highly saline water.

In addition, lingering differences in tolerance of humans may have also separated the species fundamental niches. Unlike P-horses, Khulan avoided some water points where human activity was high. Until very recently Khulan were heavily hunted [37]. Records showed that during China’s food shortages in 1960, one county alone in Xinjiang killed 6927 Khulans [38]. Hunting ceased after firearms were banned and conservation laws were enacted, but Khulan still exhibited intolerance of human activity (escape distance of > 500 m is still common), even when human activity was fairly benign as it is near ranger base stations. In contrast, P-horses showed no aversion to humans perhaps because of their association with supportive people before being reintroduced to the site and subsequently during winter corralling when interactions with human food providers is frequent. Overall, differences in ability to tolerate highly saline water and human activity have enabled the equid species to exhibit largely different fundamental niches.

Second, intrinsic differences in balancing acquisition of food and water may also play a role in separating the fundamental niches. P-horses tended to drink during the day, especially at those they preferred, whereas Khulan tended to drink at night, even at those rarely frequented by P-horses. Many truly desert-adapted species limit activity during the day to reduce heat loading and water loss [51]. Such a coping strategy normally requires a large body size to withstand high heat loading and to cope with thirst during the day. Despite their large size, P-horses were unable to behave in this way and even at noon when heat loading likely reached peak horses drank most frequently to facilitate evaporative cooling [52]. Although horses sometimes used saline water, they frequented these sites at night (NDWVIs around or below zero) presumably not to compound heat loading with salinity stress. The timing of drinking by P-horses suggests that they are not ideally suited for desert conditions. Early Silk Road travelers described “the wild horse living by the water” [53]. GPS telemetric data on P-horse movements in the Mongolian Gobi also suggested that P-horse never range far from the riparian areas [23].

In our study, Khulan drank mostly during the night. That they did so at water points infrequented by P-horses even if rarely, suggests that nocturnal watering is in part voluntary. Drinking and moving at night either while feeding or migrating would lower heat loading, reduce water loss and reduce the need to drink during the day.

Despite the propensity for the two species to display differences in their fundamental niches, especially with respect to water needs and drinking behavior, Khulan are not strictly nocturnal, or even crepuscular. They still remain active during the day, require water and thus need to drink. From the camera trap frames with individuals of both species present, it appears that a strong interspecific dominance asymmetry exists. Horse can drink where and when they want, while Khulan mostly have to wait until horses leave a water point. As illustrated in overall water use patterns (Fig 3B), both species prefer W02 with the lowest salinity and least human interference and Khulan tend to be seen there much more frequently than P-horses. However, their circadian drinking preference quickly skewed from non-selective to mostly nocturnal as P-horse escalated their visitation at W02 (Fig 5). When captured together in the same camera frame, P-horses also have priority of access and at times actively exclude Khulan from standing nearby (Table 4).

In addition, visual observations from a blind at water point W02 revealed that groups of Khulan fled when P-horses approached and in some instances P-horses even charged herds of Khulan and drove them away (Cao and Zhang, unpublished data). Physiological limitations constrain the daytime mobility of P-horses and set the stage for intense competition between the different equid species for high quality water, especially when that water is distant from potential human disturbance. Since P-horses were largely tethered to the water points during the day at this time of year because they needed to drink multiple times during a day, it would have been difficult for Khulan to gain access even if they wanted to. Such a propensity to avoid P-horse aggression is likely why Khulan visited W02 less frequently from what we would expect during both daytime and nighttime, even though W02 is further from any ranger post. That the smaller bodied Khulan can tolerate highly saline water provides them with a physiological refuge that mitigates their poorer competitive ability and helps separate the realized niches of the two species. Differences in needs and fighting abilities appear to reduce overt interspecific aggression, lessening the incidence of direct competition. Therefore, it appears that circadian differences in drinking rhythm at high quality water points emerge from a combination of the Khulan’s inferior competitive ability relative to P-horses and superior tolerance of high salinity and temperatures. But are the factors likely to be equally important in separating these equid niches?

Ultimately competition may have primacy in influencing this niche differentiation between the species rather than the physiological traits associated with differences in their fundamental niches. Initially, increases in P-horse aggressiveness may have emerged because of its need to drink repeatedly during the day [54]. Hence when challenged by Khulan, P-horses escalated contests in order to maintain control of water when most needed. That Khulan were able to forage and drink at saline sites during the day or drink at high quality water points during the night enabled them to respond to the challenges arising from the appearance of P-horses after their translocation to the KNR dessert. Similar niche divergence has been seen in two coexisting spiny mice, *Acomys cahirinus* and *A*. *russatus*. Both were nocturnal when living allopatrically, but the latter became diurnal when they became sympatric [25].

Kaczensky et al. studied the drinking behavior of P-horses and Khulan in the Mongolian Gobi, using direct observations and GPS tracking. They found that Khulan drinking showed no day-night biases [28]. Apart from differences in observational methods (e.g. direct vs. camera traps), the major factor accounting for such a divergence in temporal patterning between study sites was the size and nature of water sources. Water points in the Mongolian Gobi are much larger than in KNR thus affording multiple access and potentially enabling the two species to share one water simultaneously without strife [23]. This was apparently the case since P-horses in Mongolian Gobi showed fewer agonistic behaviors towards each other and their congeners. Similarly, Khulan showed little reaction to the presence of P-horse during simultaneous encounters [28].

Although the two equids both shared heightened needs for water in the Kalamaili desert, the species co-existed by frequenting different water points at different frequencies and at different times of day. Varying tolerances for salinity stress and human activity might have played a role allowing the species to coexist. But these conditions alone are not sufficient to explain the differences in temporal use of a common set of water points. Ultimately, differences in water needs have apparently led to differences in the value of consuming water, which in turn have intensified potential competition between the species. Direct competition, however, is minimized because Khulan physiology allows it to adopt an alternative and energetically efficient strategy of nocturnal drinking and daytime feeding that is unavailable for P-horse. Although both species can co-exist because of realized niche segregation, costs associated with competition are not eliminated.

## Supporting Information

S1 Fig Pie plot showing the number of trapping events and percentages by taxonomic categories. (PDF)

S2 Fig Jitter scatter plots showing two equids’ daily trapping events (number of daily water visits) and partial residuals in relation to water points’ salinities and shortest distances to monitoring stations. (PDF)

S3 Fig Jitter and violin plots showing the distributions of two equids’ Normalized Daytime Water Visit Indices (NDWVI) at four water points. (PDF)

S1 Table Results of partial correction tests of two equids’ Normalized Daytime Water Visit Indices (NDWVI) in relation to the environmental and interspecific factors. (PDF)
