# Supplementary figures and images for: Water Use Patterns of Sympatric Przewalski’s Horse and Khulan: Interspecific Comparison Reveals Niche Differences
Source: PLoS One. 2015 Jul 10;10(7):e0132094. doi: 10.1371/journal.pone.0132094 (PMC4498657; doi:10.1371/journal.pone.0132094)

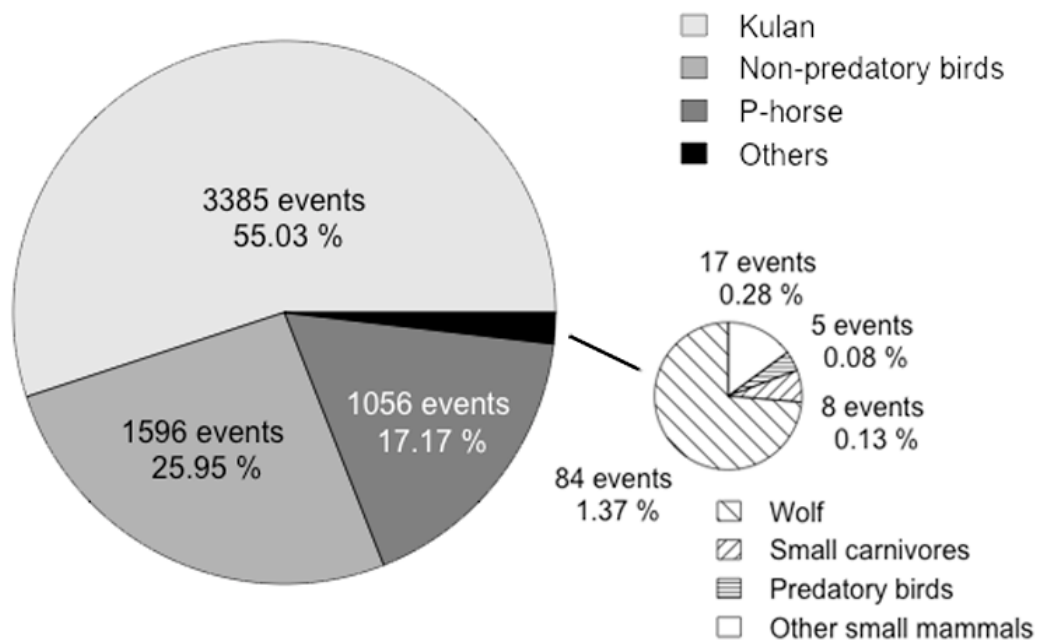

**S1 Fig. Pie plot showing the number of trapping events and percentages by taxonomic categories.**

Supplement: S1 Fig — (PDF) [file pone.0132094.s001.pdf]
