# Supplementary material for: Water Use Patterns of Sympatric Przewalski’s Horse and Khulan: Interspecific Comparison Reveals Niche Differences
Source: PLoS One. 2015 Jul 10;10(7):e0132094. doi: 10.1371/journal.pone.0132094 (PMC4498657; doi:10.1371/journal.pone.0132094)
Supplement: S2 Fig — (PDF) [file pone.0132094.s002.pdf]

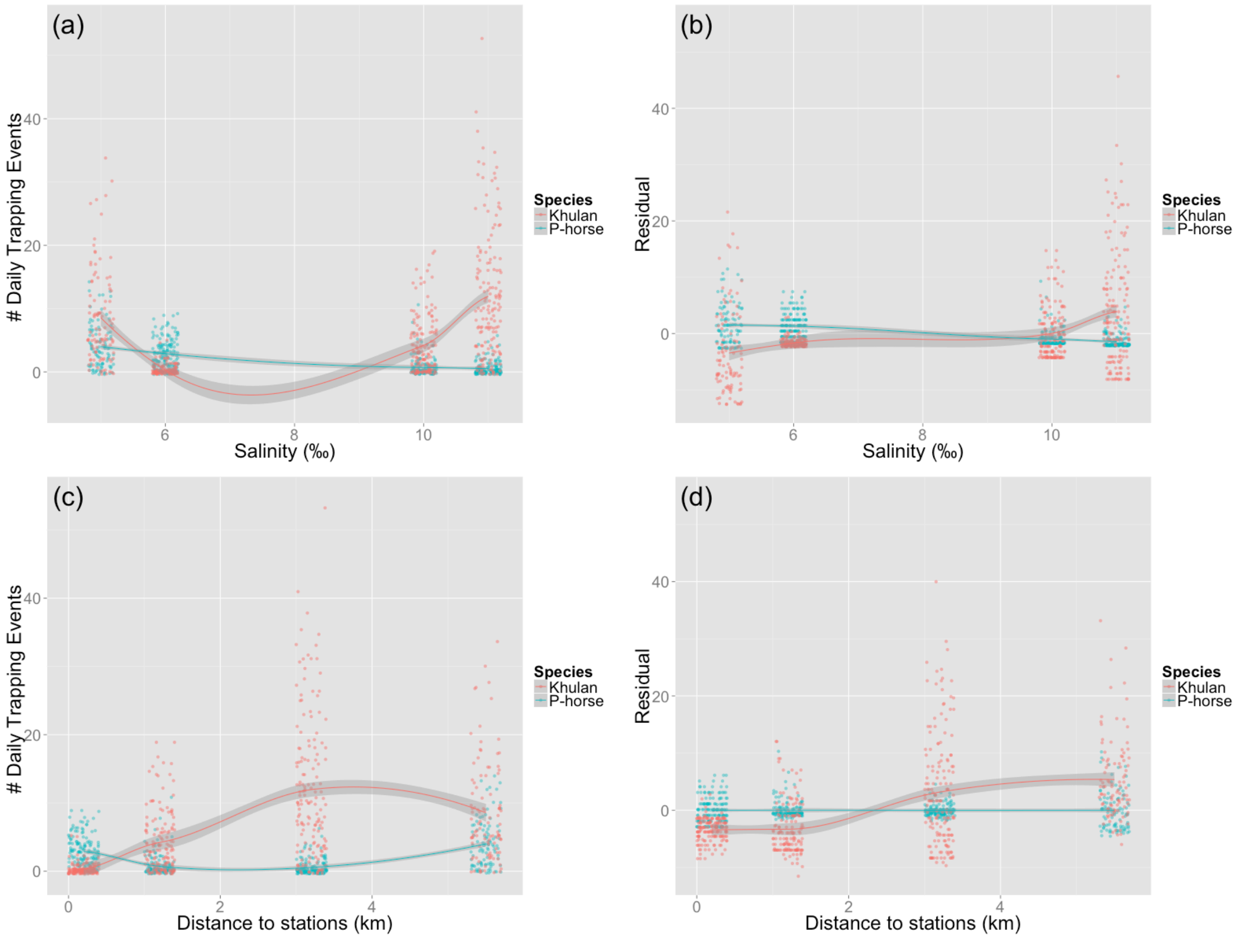

S2 Fig. Jitter scatter plots showing two equids' daily trapping events (number of daily water visits) and partial residuals in relation to water points' salinities (a, b) and shortest distances to monitoring stations (c, d). P-horses and Khulans were highlighted in cyan and orange respectively. Number of daily water visits (dependent variable) of one equid was fitted by that of the other equid, as well as measures of salinity and distance to station (covariates), using linear regression models. Residuals and regression coefficient of each covariate were obtained to calculate partial residuals, which were plotted against salinity (b) and distance to water (d) respectively. The trend lines were fitted with the Loess smoothing functions with gray bands of 95% Confidence Interval (CI).
