# Supplementary material for: Water Use Patterns of Sympatric Przewalski’s Horse and Khulan: Interspecific Comparison Reveals Niche Differences
Source: PLoS One. 2015 Jul 10;10(7):e0132094. doi: 10.1371/journal.pone.0132094 (PMC4498657; doi:10.1371/journal.pone.0132094)
Supplement: S3 Fig — (PDF) [file pone.0132094.s003.pdf]

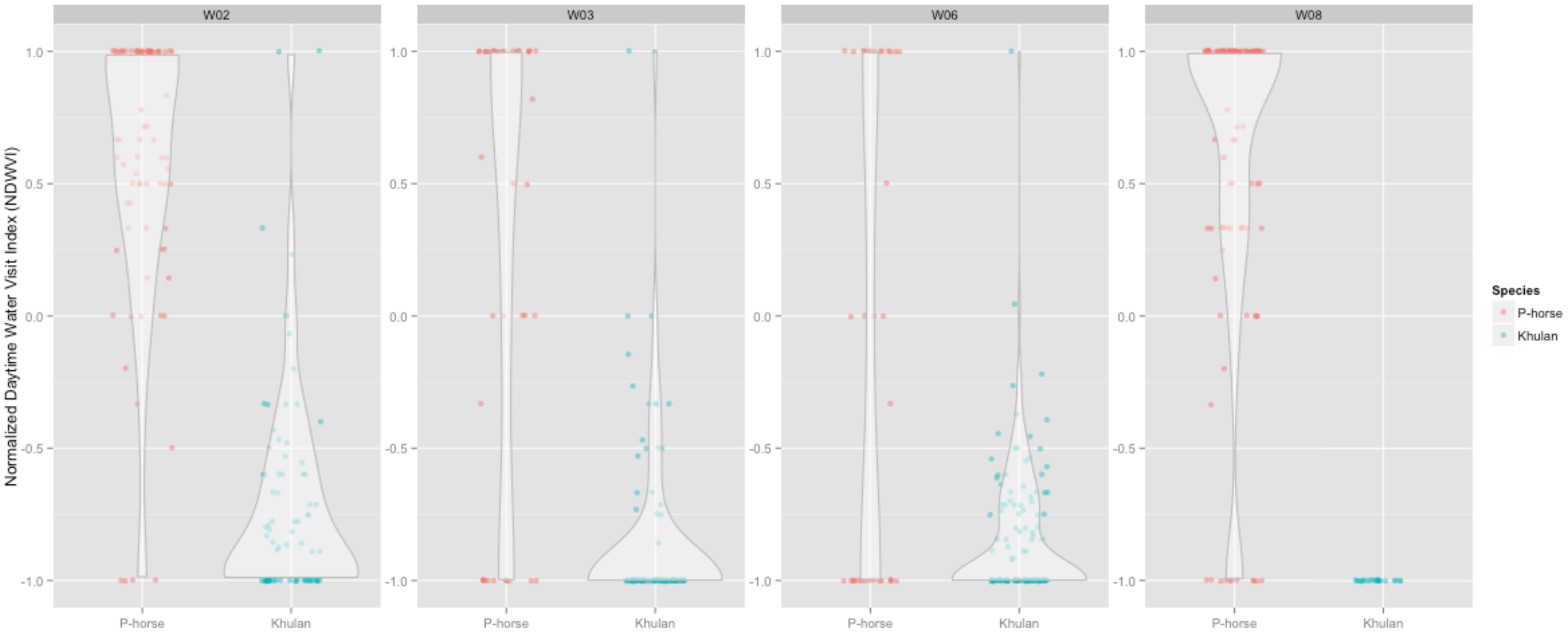

S3 Fig. Jitter and violin plots showing the distributions of two equids' Normalized Daytime Water Visit Indices (NDWVI) at four water points.
