# Supplementary material for: Water Use Patterns of Sympatric Przewalski’s Horse and Khulan: Interspecific Comparison Reveals Niche Differences
Source: PLoS One. 2015 Jul 10;10(7):e0132094. doi: 10.1371/journal.pone.0132094 (PMC4498657; doi:10.1371/journal.pone.0132094)
Supplement: S1 Table — (PDF) [file pone.0132094.s004.pdf]

S1 Table. Results of partial correction tests of two equids' Normalized Daytime Water Visit Indices (NDWVI) in relation to the environmental and interspecific factors.

	Salinity		Distance		P-horse		Khulan	
	CC[a]	*P*	CC	*P*	CC	*P*	CC	*P*
**Salinity**	1	0						
**Distance**	-0.523	2.94E-14	1	0				
**P-horse**	-0.217	0.006	0	0.999	1	0		
**Khulan**	0.061	0.453	0.297	1.21E-4	0.099	0.217	1	0

a. Pearson's Correlation Coefficient;

b. Strength of Corrlation: <0.1 zero correlation; 0.1-0.3 weak correlation; 0.4-0.6 moderate correlation; 0.7-0.9 strong correlation; >0.9 perfect correlation;

c. Significance level after Bonferroni correction: '***' <0.0001; '**' <0.001; '*' <0.005.
